# Optimizing Inorganic Carbon Sequestration and Crop Yield With Wollastonite Soil Amendment in a Microplot Study

**DOI:** 10.3389/fpls.2020.01012

**Published:** 2020-07-03

**Authors:** Fatima Haque, Rafael M. Santos, Yi Wai Chiang

**Affiliations:** School of Engineering, University of Guelph, Guelph, ON, Canada

**Keywords:** enhanced weathering, soil amendment, field crops, plant growth, CO_2_ sequestration, inorganic carbonates

## Abstract

Carbon dioxide (CO_2_) is a major greenhouse gas, and its concentration in the atmosphere is increasing continuously, hence there is an urgent need to reduce its level in the atmosphere. Soils offer a large natural sink to store CO_2_. This study focuses on sequestering CO_2_ in the agricultural soils as inorganic carbon, which can be accomplished by adding alkaline-earth silicates. Wollastonite is used in this study as a soil amendment, to sequester CO_2_ *via* the geochemical route of mineral carbonation. The first objective of the present study was to evaluate the effect of mixing a wide range of dosages of wollastonite, as a soil amendment, on the growth performance of two leguminous plants frequently used in agricultural sector: soybean and alfalfa. The plants were grown with different wollastonite dosages (3–20 kg·m^−2^ for soybean and 3–40 kg·m^−2^ for alfalfa), for a duration of 14 weeks in a microplot experiment in Ontario, Canada. The second objective was to find evidence of enhanced weathering of wollastonite in soil, in addition to the augmentation of inorganic carbon content in soil. For this, mineralogical assessment of the soils was performed using XRD and SEM-EDS analyses. Wollastonite increased the soybean yield by two-fold in the plot amended with 10 kg·m^−2^. At all dosages, wollastonite increased the alfalfa growth in terms of height and above-ground biomass dry weight, as well as root biomass. The rate of CO_2_ sequestration, at optimum wollastonite dosage, reached 0.08 kg CO_2_·m^−2^·month^−1^. XRD and SEM-EDS analyses indicated accumulation of calcite in wollastonite-amended soil and formation of other weathering products. The results obtained from this study help to understand the impact of wollastonite soil amendment on agronomy, and will aid in implementing such negative emissions technology by informing farmers and industry alike that the use of wollastonite contributes toward global climate change mitigation while supporting crop yield. The findings of this study add to the existing body of knowledge on enhanced weathering as an atmospheric CO_2_ removal technology, providing further evidence that wollastonite weathering in agricultural soils can lead to significant capacity for CO_2_ sequestration as inorganic carbon, while concurrently promoting plant growth.

## Introduction

Agriculture can contribute to greenhouse gas mitigation, either *via* photosynthesis and the organic carbon cycle, or *via* terrestrial enhanced weathering of alkaline-earth silicates, also known as mineral carbonation (Hartmann et al., 2013; Zomer et al., 2017). While photosynthesis contributes towards increasing carbon storage in biomass and soil organic matter, terrestrial enhanced weathering of calcium and magnesium silicate rocks and the subsequent precipitation of calcium or magnesium carbonates in the soil can additionally lead to augmentation of inorganic carbon content of soils and the underlying formations (Moosdorf et al., 2014; Machmuller et al., 2015). Several independent research groups have recently reported on the increased inorganic carbon content of soils amended with alkaline-earth minerals (Renforth et al., 2009; Renforth et al., 2011; Manning et al., 2013; Washbourne et al., 2015; Amann et al., 2020). Hence, using alkaline-earth mineral soil amendments to grow plants has the potential to mitigate atmospheric CO_2_.

The addition of certain alkaline-earth minerals to land has been shown to enhance soil quality and plant productivity, especially in nutrient poor and highly weathered acidic soil (Jones and Handreck, 1965; Mitani and Ma, 2005; Silva et al., 2005; Keller et al., 2012; Haynes, 2014; Meena et al., 2014). Using alkaline-earth minerals as a soil amendment not only contributes towards soil inorganic carbon accumulation (and thus CO_2_ sequestration), but also amends soil chemical properties potentially resulting in improved soil fertility. Some agricultural soils are characterized by low available calcium (Ca) and high available aluminum (Al) content; as a result, plant root growth will be impaired, and water and nutrients uptake by plants will be affected (Haque et al., 2019a). Van Straaten (2006) has evaluated the effect of multi-nutrient silicate rock fertilizers on the movement of nutrient around the root surface as well as the biochemical processes, finding that it has the potential to supply the soils with macronutrients (N, P, K) and micronutrients (especially Ca and Mg).

Wollastonite (nominally CaSiO_3_, but also found in association with other minerals, such as diopside (CaMgSi_2_O_6_)) is a Ca-rich mineral. Wollastonite, as with other alkaline-earth silicates, undergoes mineral carbonation reaction when in an aqueous environment saturated with CO_2_, though the rate of reaction (both silicate dissolution and carbonate precipitation) varies depending on geochemical conditions, such as the CO_2_ partial pressure and the pH (Lackner, 2003; Huijgen et al., 2006; Manning and Renforth, 2013). Equations (1)–(3) show how carbon sequestration *via* wollastonite weathering occurs (Hangx and Spiers, 2009).

(1) $$\text{C}{\text{O}}_{2}\;\text{dissolution}:\;2\text{C}{\text{O}}_{2(\text{g})}+2{\text{H}}_{2}{\text{O}}_{\left(1\right)}↔2{\text{H}}_{2}\text{C}{\text{O}}_{3(\text{aq})}↔2\text{HC}{\text{O}}_{3^{-}}+2{\text{H}}^{+}$$

(2) $$\text{Wollastonite}\;\text{dissolution}:\;\text{CaSi}{\text{O}}_{3(\text{S})}+2{\text{H}}^{+}\to \text{C}{\text{a}}^{2+}+{\text{H}}_{2}{\text{O}}_{\left(1\right)}+\text{Si}{\text{O}}_{2(\text{S})}$$

(3) $$\text{Calcium}\;\text{carbonate}\;\text{precipitation}:\;\text{C}{\text{a}}^{2+}+2\;\text{HC}{\text{O}}_{3^{-}}\to \text{CaC}{\text{O}}_{3(\text{S})}↓+{\text{H}}_{2}{\text{O}}_{\left(1\right)}+\text{C}{\text{O}}_{2(\text{g})}$$

These reactions show that wollastonite has the potential to sequester carbon dioxide (CO_2_) from the surrounding environment as well. If this process occurs in the agricultural soil, it would offer an attractive sequestration method. Among a wide variety of natural alkaline-earth silicates for the terrestrial weathering process, wollastonite is one of the most promising candidates because of its simple chemistry, high weathering rate, and the ease of production of carbonated products due to the weaker bonding of Ca ions to the silica matrix (Palandri and Kharaka, 2004; Schott et al., 2012). Wollastonite is widely distributed around the world, occurring in China, Finland, India, Mexico, Spain, Canada, and the U.S., with a reserve size exceeding 100 million tons (Brioche, 2018). The main source of wollastonite in Ontario, Canada (where this study was conducted), is a surface mine operated by Canadian Wollastonite in the village of Seeley's Bay, located 30 km north of the city of Kingston.

In our previous study, Haque et al. (2019c) determined the role of plants: legumes (green bean) and non-legumes (corn), on wollastonite weathering and inorganic carbonate formation in soil. Legumes produce root nodules, which aid in nitrogen fixation, hence releasing protons into the soil (Hardarson, 1993; Danyal et al., 2016). The protons released facilitated wollastonite weathering and resulted in increased release of calcium ions in the soil, thus capturing more dissolved atmospheric/soil CO_2_, leading to increased formation of calcium carbonate in the soil. Non-legume plant (corn) grew better in the wollastonite-amended soil, but the inorganic carbonate accumulation, in this case, was less compared to that of the legume plant (Haque et al., 2019c). Hence, in the present study, leguminous plants were selected to further investigate the effect of wollastonite on the growth performance of two plant species commonly used in agriculture: a high-value agricultural crop, soybean (*Glycine max*), and a cover/forage crop, alfalfa (*Medicago sativa*). In the field, soybean is usually grown in the summer and post-harvest the field can be covered with alfalfa.

Wollastonite weathering under laboratory conditions is well documented (Huijgen et al., 2006), however, the only experimental data under northern hemisphere cropped conditions is available in our previous pot study (Haque et al., 2019c), and in our recently published field study (Haque et al., 2020). In the latter, we analyzed the soil from two commercial fields in Ontario, where wollastonite was being used as a soil mineralizer, to identify evidence of soil inorganic carbon accumulation (as pedogenic carbonates), and also conducted a field experiment to grow soybean under three different dosages of wollastonite. At a field-scale, the main limitation is the use of low application rates of wollastonite. As there is a limited study on the use of wollastonite as a soil amendment, commercial producers do not consider testing higher application rates. As a result of the low application rates, as well as the large area of fields, the main challenge is to separate the wollastonite grains and weathering products from the soil to study morphological and mineralogical changes. Therefore, to provide more evidence on the effect of wollastonite on plant growth, as well as wollastonite carbonation in soil under ambient conditions, this present microplot study was carried out. Also, the plants selected for this study are two common plants (soybeans and alfalfa) used in the agricultural sector; hence, with the results established from this microplot study, the growers can make an informed decision to apply wollastonite on their field. The first objective of this study was to evaluate the benefit of mixing a wide range of dosages of an alkaline-earth silicate, wollastonite, as a soil amendment, on the growth performance of the plants in terms of above-ground biomass dry weight, root biomass, stem width, leaf blade width, and plant height. For soybean, the weight of the pods was also measured to determine the effect on the yield. The second objective was to find evidence of enhanced weathering of wollastonite in soil. In addition to determining the inorganic carbon content in the soil, mineralogical assessment of the soils was performed using non-destructive techniques useful in characterizing mineral weathering and carbonation (Haque et al., 2019b), namely XRD and SEM analysis. The results obtained from this study help to understand the impact of wollastonite soil amendment on agronomy, and will aid in implementing such negative emissions technology.

## Materials and Methods

### Soil and Plant Selection

The soil, used to set up the microplot on the rooftop, was collected from a commercial agricultural field located in Woodstock, southwestern Ontario, Canada (43°08'57.7”N 80°37'29.8”W, EL 247m). The soil classification is identified as sandy loam (gravel 11 g/kg, sand 551 g/kg, silt 295 g/kg, clay 155 g/kg) with a pH of 6.63 and 32 g/kg dry organic matter content. Its taxonomic classification, based on historical soil surveys of the area (Mozuraitis and Hagarty, 1996), is orthic melanic brunisol, with soils from this area having been formed on glacial till (Webber and Hoffman, 1967), characterized as rapidly drained with high water conductivity, and being non-stony, sandy, mixed, mildly alkaline, and strongly calcareous. Refer to **Table S1** for more information.

Since soybean was grown on this particular farm, and it is a major global and regional crop, it was selected as the agricultural crop for this study. A leguminous cover crop was selected to take advantage of the benefits that nitrogen-fixation activity in root nodules was found in our prior study to bring to wollastonite weathering (Haque et al., 2019c). To this end, alfalfa, as a widely used leguminous forage crop, was selected. Soybean (*G. max*) seeds coated with *Bradyrhizobium japonicum*, and alfalfa (*M. sativa*) seeds coated with *Sinorhizobium meliloti*, were used; the seeds were coated with the aforementioned bacteria for their legume-root nodulating and nitrogen-fixing abilities.

### Microplot Setup

The experimental microplot was set up at the Thornbrough building rooftop in the University of Guelph, Ontario, Canada. Soybean plants were grown with four different wollastonite-in-soil dosages, containing 1.5, 5, 7.5, and 10 wt.% mineral soil amendment (MSA), along with a control plot of the unamended soil. These dosages are equivalent to 3 kg·m^−2^ to 20 kg·m^−2^ based on 0.15 m soil depth, which is the usual tillage depth employed at the fields. Alfalfa plants were grown with six different wollastonite-in-soil dosages, containing 1.5, 5, 7.5, 10, 15, and 20 wt.% MSA, along with a control plot of unamended soil. Wollastonite was tumbled with soil in large buckets for blending, with the aim being to achieve good mixing but with minimal disruption to soil texture.

Testing a wide range of wollastonite dosage on experimental plots will help to understand which dosage range is best suited for field application, and determine an upper limit beyond which plant and mineral weathering benefits decline. A higher dosage was investigated to study the alfalfa since as a winter-hardy cover crop, it could be used in fields that decide to apply wollastonite at larger dosages in late-Fall post-harvest, just prior to alfalfa seeding. Moreover, at the field scale, the highest suitable amendment dosage is not intended to be used in a single application, but could be thought of as the cumulative of several applications over multiple crop cycles.

**Figure 1:**
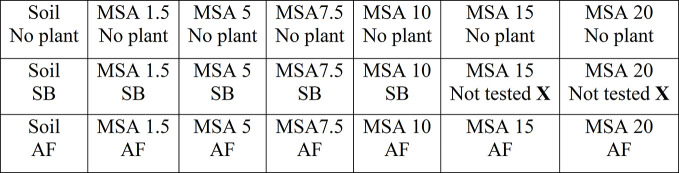
Schematic representation of the experimental setup. The first row represents the plots with no plants, and first column represents the control plot using unamended soil as-received. “MSA” denotes “Mineral soil amendment”, “MSA 1.5” to “MSA 20” denotes MSA with 1.5 to 20 wt.% wollastonite amendment, “SB” denotes soybean and “AF” denotes alfalfa.

Plots of various MSA compositions (1.5 to 20 wt.%) without any plants were also maintained to check for the wollastonite weathering and carbonate accumulation under uncropped conditions, hence enabling distinction of plant effects on the weathering processes versus soil and ambient effects. Each microplot, with a dimension of 0.6 m length, 1.2 m width, and 0.15 m depth, was filled with soil or MSA. No other chemical, mineral or organic amendments (e.g. solid or liquid fertilizers, limestone/dolomite, manure/compost, etc.) were used in these experiments, as the field soil used was considered to be of adequate fertility for the purpose of this research. The plots were open to drainage to emulate the setup as close as possible to the field conditions. At the start of the experiment, the soils were supplied with adequate tap water, and later depended on rain water as the source of water supply. Daily climatic data from this study is reported in the Supplementary Material (**Figure S1**). The maximum, minimum, and mean temperatures recorded during the experimental run are 25.7, 12.6, and 19.2°C, respectively. The schematic of the experimental set up is given in **Figure 1** and **Figure S1C**. The experiments ran for 14 weeks, from late June to early October, 2018. At the end of the experiment, the soil from each plot was sampled, using a soil core sampler (0.013-m diameter) at five different points radially distributed within each microplot and down to full depth (0.15 m); cores were thoroughly hand-blended, air dried, and sieved through 200 µm mesh prior to all soil analyses.

### Plant Growth Trials

The plant growth and development were analyzed based on the development stages. At the end of the experimental run, after 14 weeks, the plants were harvested by cutting them just above the soil level. The number of plants recovered from each microplot was: 30 soybean plants, and 15 alfalfa plants. The main growth parameters recorded were plant height, stem width, leaf blade width, root dry biomass, and above-ground biomass dry weight. The root dry biomass of each plant was determined by separating the roots from the soil by washing with sufficient water and drying the sample at 80°C for 48 h. Above-ground biomass dry weight of each plant was determined after drying the sample in a drying oven at 80°C for 48 h (Jones et al., 2009). All such measurements reported in the results section were averaged among all collected plants from each microplot, and standard deviations values were calculated. The yield of the soybean pods collected from the plants at week 14 was determined from the combined mass of pods from each subplot with each microplot. Subplots contained 10 soybean plants. Thus the yield was averaged between the three subplots in each microplot, and standard deviation calculated.

### Chemical Analyses

The pH of the samples was determined using a 1:5 ratio of soil and 0.01 M CaCl_2_ solution, followed by 30 min shaking and 1 h settling time before taking the pH measurement of the clear supernatant (Pansu and Gautheyrou, 2007).

The carbonate content (calculated as g CaCO_3_·(kg soil)^−1^) was determined by using a calcimeter. Soil samples were suspended in MiliQ water (5 g in 20 ml), to which 7 ml of 4M HCl was added within in a sealed Erlenmeyer flask connected to a graduated water-filled manometer-style column that recorded the released CO_2_ volume (Eijkelkamp Calcimeter 08.53) (Chen et al., 2015). The amount of CaCO_3_ accumulated in the soil as the result of the sequestration of the dissolved CO_2_ present in the soil pore-water by the calcium ions dissociated from wollastonite is calculated using Equation (4). The CaCO_3Initial_ value accounts for the carbonate content added to the soil as the result of wollastonite application (which contains small amounts of naturally present calcite) together with the initial CaCO_3_ content in the untreated soil.

(4) $$\text{CaC}{\text{O}}_{3}\;\text{accumulation}\;\left(\frac{\text{g}}{\text{kg}}\right)=\text{CaC}{\text{O}}_{3\text{Final}}(\frac{\text{g}}{\text{kg}})-\text{CaC}{\text{O}}_{3\text{Initial}}(\frac{\text{g}}{\text{kg}})$$

The pH and calcimetry analyses were made in triplicates and mean results reported herein have been represented along with standard deviations. The data were statistically analyzed using one-way analysis of variance (ANOVA) along with Tukey test. P < 0.05 was used as the limit for statistical significance. Data analysis was done using IBM SPSS Statistics 26 software.

### Mineralogical and Microstructural Analyses

The mineralogy of the soils and mineral was determined by X-ray diffraction (XRD, Panalytical Empyrean) operated with Cu Kα radiation at 45 kV and 40 mA. The diffraction patterns were collected over a 2θ range of 5–70° (Gineika et al., 2019) and the crystalline phases were identified using the software HighScore Plus (Malvern Panalytical). The morphology, structure, and chemical composition of samples were analyzed using a scanning electron microscope (SEM, FEI Inspect S50) equipped with an energy-dispersive X-ray spectrometer (EDS, Oxford X-Max20 SSD).

### Wollastonite Characteristics

Wollastonite for the study was sourced from Canadian Wollastonite's Ontario mine (44°27'30''N, 76°15' 20''W, Sangster (1998)). Analysis using X-ray diffraction (XRD) showed that the main mineral phases included wollastonite (CaSiO_3_) and diopside (CaMgSi_2_O_6_) (**Figure S2**). The fine wollastonite consisted of 49.0 wt.% wollastonite, 20.4 wt.% diopside, and the remainder free SiO_2_ and minor silicates, aluminates and sulfates. The inherent calcite (CaCO_3_) amount of 3.8 wt.% was determined by using the calcimeter.

Elemental composition of the soil was quantified by Wavelength Dispersive X-Ray Fluorescence (WDXRF, Malvern Panalytical Zetium). Duplicate samples, in loose powder form, were analyzed for 20 min using standardless Omniam method, under helium and at 1 kW power, and concentrations were calculated as oxides. The average sum before normalization was 88.3 wt.%, with the balance being porosity and undetectable light elements (H, C, O, N). The average concentrations of the detected oxides present in amounts greater than 0.10 wt.%, normalized to 100% were: 52.5 wt.% SiO_2_, 29.8 wt.% CaO, 4.63 wt.% MgO, 4.04 wt.% Al_2_O_3_, 3.17 wt.% Fe_2_O_3_, 1.61 wt.% K_2_O, 1.57 wt.% Na_2_O, 1.30 wt.% SO_3_, 0.74 wt.% P_2_O_5_, 0.24 TiO_2_; 0.19 SrO.

The particle size distribution of wollastonite was determined by wet laser diffraction (Malvern Mastersizer SM), and the surface weighted (Sauter) mean diameter (D [3,2]) was found to be 4.37 ± 0.06 µm and 90% of particles by volume (D_90_) were less than 63.7 µm (**Figure S3**). The multipoint BET (Brunauer, Emmett and Teller) specific surface area was determined to be 3.476 m^2^·g^−1^. This was performed by a physisorption analyzer (Quantachrome Autosorb iQ), using N_2_ adsorption at 77K on samples previously degassed under vacuum, consecutively at 120°C (30 min soaking time) and 350°C (300 min soaking time).

## Results and Discussion

### Effect of Mineral Amendment on Plant Growth

**Figure 2:**
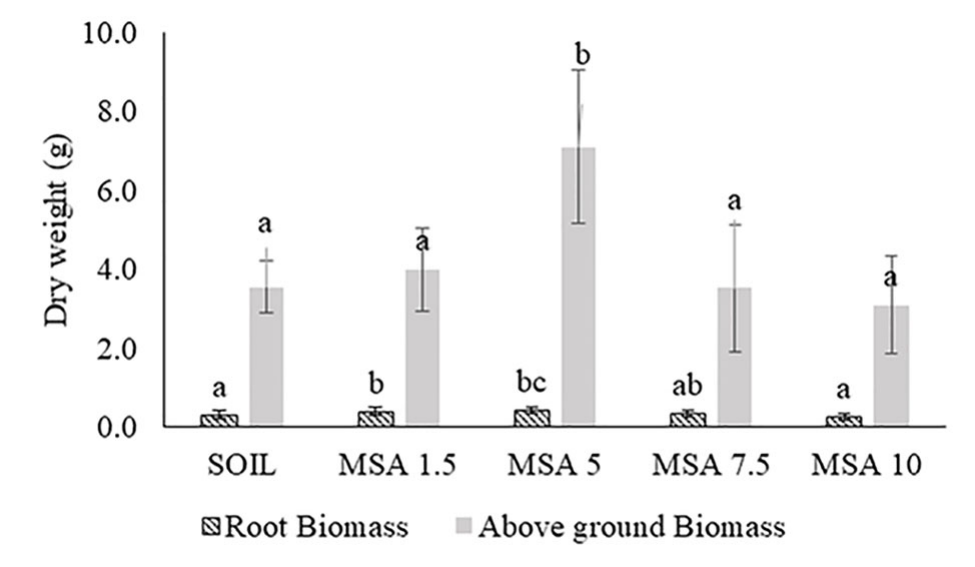
Variation in the soybean: a) root biomass, and b) above-ground biomass dry weight, with different dosages (wt.% in soil) of wollastonite mineral soil amendment (MSA). In all cases, n = 30 and statistically analyzed using Tukey test.

**Figure 2** shows the variation in the soybean root biomass and above-ground dry biomass, when grown with different wollastonite dosages, using a sample size (n) of 30 plants. The plant height data is given in **Figure S4**. The soybean trials showed that the plants performed best in the 5 wt.% wollastonite-amended soil, with the plants exhibiting increased plant height (8.1% higher), and greater root biomass (32.5% greater) and above-ground biomass dry weight (twofold greater), in comparison to those grown in the control plot as well as showed a statistical difference (p <0.05). In all treatments, the roots had reached the base of the growth containers and exhibited healthy root biomass. There was no significant change in the stem and leaf blade widths for the various treatments (**Table S2** in the Supplementary Material).

**Figure 3:**
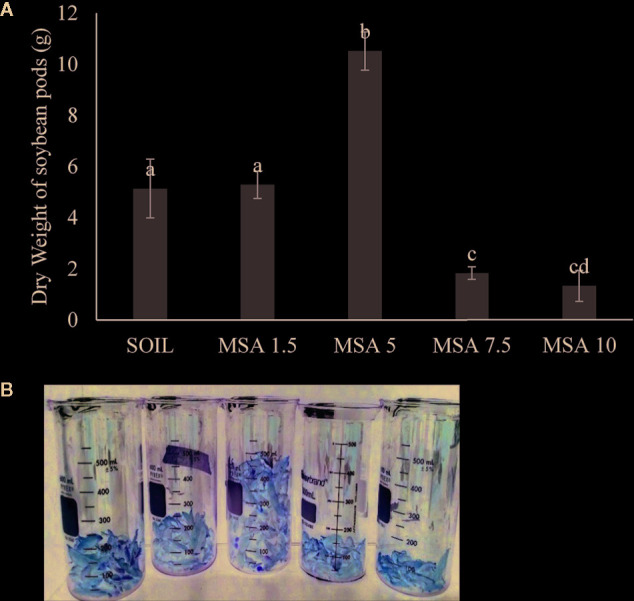
**(A)** Variation in the weight of the soybean pods with increasing wollastonite dosage analyzed per subplot of 10 plants. **(B)** Picture of the soybean pods from each microplot of 30 plants (left to right: SOIL, MSA 1.5, MSA 5, MSA 7.5, MSA 10).

The 5 wt.% wollastonite-amended soil showed the highest soybean yield (twofold greater), as seen in **Figure 3** and **Figure S5**. The yield decreased for the 7.5 and 10 wt.% MSA microplots. At the end of the growth trial, the pH of the MSA 7.5 and MSA 10 soils was 7.43 ± 0.04 and 7.76 ± 0.05, respectively, whereas for the MSA 5 soil the pH was 7.11 ± 0.03 (**Figure S6A**). The suitable pH range for soybean is 6.5–7.0, as at higher pH, the supply of bioavailable nutrients to the plant is disrupted (Fageria and Baligar, 1999), which can be one of the reasons why the yield of soybean is low in case of the microplots with the two highest wollastonite dosages. Even though the yield in case of MSA 7.5 is low in comparison to the control (unamended soil), there is no significant difference (p >0.05) in the plant height, root biomass, and above-ground biomass dry weight (**Figure 2** and **Figure S4**), which implies the plant grew well, but was less efficient in its reproductive stage. For MSA 10 microplot, root biomass was lower by 14.7% as compared to the control, and poor root biomass density can be responsible for shorter plant height (18.2%) and lower above-ground biomass dry weight (12.7%). Overall, all tested wollastonite dosages did not result in negative growth performance, i.e., zero seedling germination or plant senescence before the end of the growth trial. This implies that an appropriate amount of wollastonite, when added to the soil, supports good plant growth.

**Figure 4:**
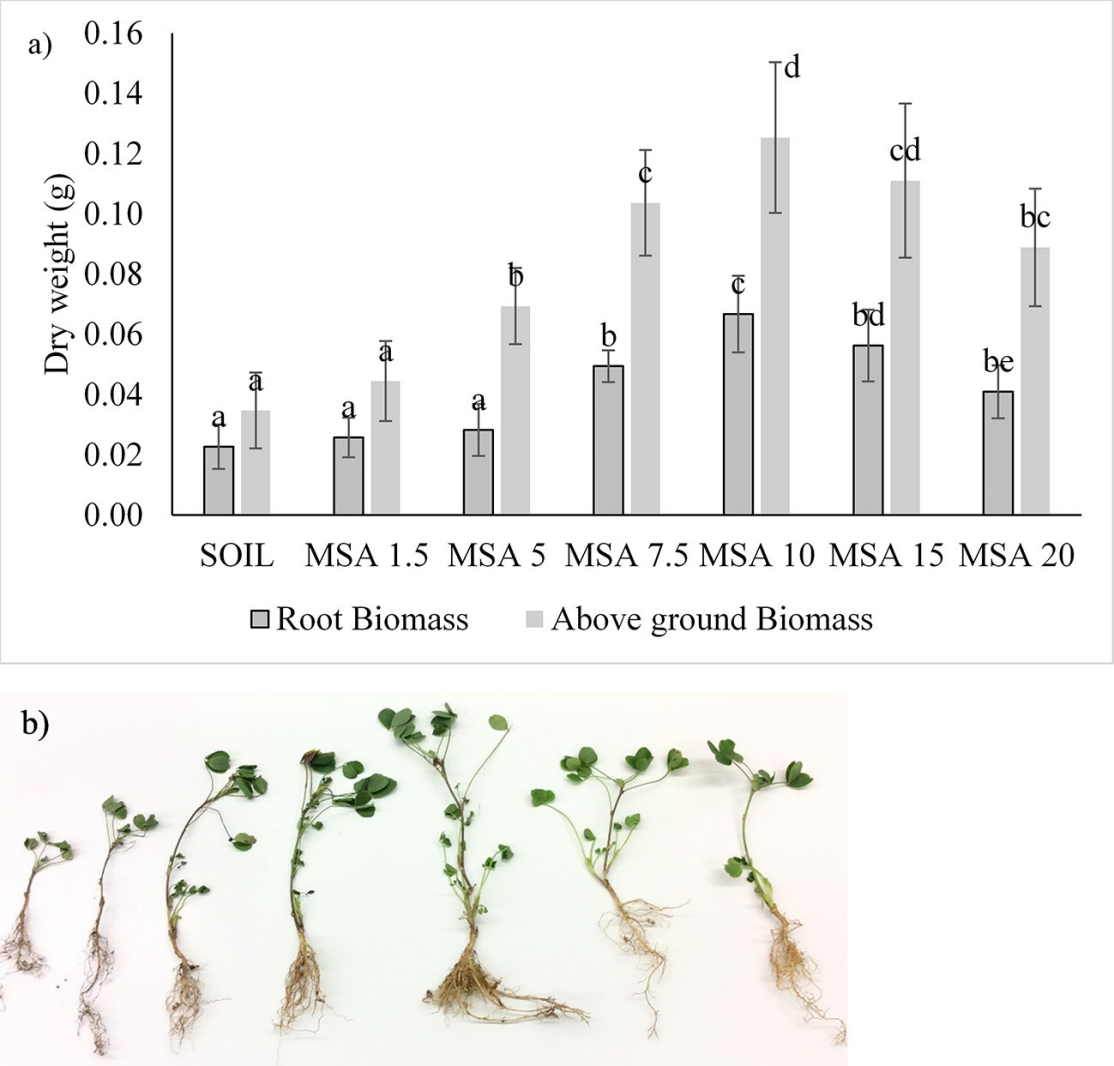
Variation in the alfalfa: **(A)** root biomass and above-ground biomass dry weight, with different dosages (wt.% in soil) of wollastonite mineral soil amendment (MSA). In all cases, n = 15 and statistically analyzed using Tukey test. **(B)** picture of the alfalfa grown in various treatment (left to right: SOIL, MSA 1.5, MSA 5, MSA 7.5, MSA 10, MSA 15, MSA 20).

The growth response of the alfalfa to wollastonite amendment is shown in **Figure 4** (n = 15). Overall, the growth of alfalfa was better than the control in all treatments. In contrast to the soybean trials, growth of alfalfa was best with 10 wt.% wollastonite-amended soil. At the end of the 14-week growth period, the alfalfa grown in MSA 10 was taller in height by threefold (**Figure S7**), possessed higher above-ground dry weight (by 3.6-fold), and had threefold greater root biomass in comparison to the control (p <0.05). Amending the alfalfa microplots with wollastonite increased the soil pH, as seen in **Figures S6B** and **S7B**. The pH of the control soil plot was 6.42 ± 0.05, and that of the 20 wt.% MSA plot was 8.09 ± 0.04, which implies that higher wollastonite dosage can be used for plants preferring alkaline soil for their growth, for which alfalfa can be a candidate. Alfalfa is a cover crop that is usually sown after the growing season, therefore this cover crop would be compatible with applying a higher wollastonite dosage that could help with speeding up wollastonite incorporation into the soil. Then in the upcoming season, when other agricultural crops will be planted, the soil will be well mineralized with wollastonite as a result of the cover crop-timed application.

Wollastonite-amended soil promoted good plant growth, thus confirming its potential to be used as a soil amendment in the agronomic sector. Canadian Wollastonite and its distributors have already been marketing wollastonite to farmers in Ontario and beyond, and assessing the effects and fate of wollastonite in commercial farms is also part of our current research. In the present study, increased soybean yield in wollastonite-amended soil, as well as healthier alfalfa growth, shows the positive effects of this alkaline-earth mineral. In addition to the aboveground plant growth, root biomass also showed a positive response to wollastonite addition. Visual inspection of the roots showed no observable signs of swollen root tips, or root browning. Root nodules formed, thus indicating the unaltered activity of rhizobium bacteria in the wollastonite-containing soil environment. These positive responses of the growth performance may be due to the release of silica into the soil (Equation (2)), which accumulates in certain plants in the form of phytogenic silica (Keller et al., 2012). Si is known to offer numerous benefits to plant growth including better yield and quality, nitrogen fixation, and alleviate the abiotic and biotic stress as a result of extreme temperatures, metal toxicity, salinity or drought (Ma, 2004; Mitani and Ma, 2005; Van Bockhaven et al., 2013). Coskun et al. (2019) provides a critical review on the mechanism of how Si can benefit the plant growth. Guntzer et al. (2012) reports that soybean plants are Si accumulators, which supports the positive growth of soybean plants in wollastonite-amended soil. Bélanger et al. (2016) offers evidence on the transport and accumulation of Si in the roots and leaves of soybean cultivars. The plant-available form of silicon in the soil is mainly as monomeric silicic acid (H_4_SiO_4_) (Dietzel, 2002), which is the hydrated form of SiO_2_, thus readily available when wollastonite weathers in soil. Hodson et al. (2005) described the high-density accumulation of phytogenic silica in the roots and shoots of soybeans and alfalfa, which explains the taller and denser root biomass observed in case of both the plant species studied in this work.

In the agriculture sector, wollastonite can be used as a liming agent, as it helps in increasing the soil pH (reducing soil acidity) by adding calcium and magnesium, thus reducing aluminum and manganese solubility in the soil (Osman, 2012; Goulding, 2016). It can be applied using the same broadcaster as the traditional lime spreader. Fertilizing the soil with Si is known to improve the yield of rice as well as sugarcane, especially in Si-deficient oxisol soils (Savant et al., 1999; Korndörfer and Lepsch, 2001; Keeping and Meyer, 2006). In fact, using wollastonite for Si fertilization of soil leads to Si accumulation in the aerial parts of the sugarcane (Rodrigues, 1997). Hence, application of wollastonite in agricultural fields is a known practice; however, using wollastonite for sequestering carbon *via* terrestrial enhanced weathering is not well reported. In our recent study (Haque et al., 2020), a field-scale wollastonite amendment experiment was conducted at a commercial soybean farm, for verification of CO_2_ sequestration and effects on crop yield at field conditions. Soils at two additional farms (leafy vegetables, and potato) that had voluntarily used wollastonite amendment for one or more crop cycles were also studied for evidence of CO_2_ sequestration. This study showed that application of wollastonite resulted in pedogenic carbonate accumulation, proportional to time since, and rate of, mineral amendment.

World reserves of wollastonite exceed 100 million tons, and in Ontario, the nearest mine is located in Kingston (Brioche, 2018). Hence, the availability and location of the alkaline-earth mineral are also important to determine its feasibility as a mineral soil amendment. Wollastonite was used in this study because it is available in Ontario, Canada. Similarly, across the world, different types of alkaline-earth minerals can be used based on their availability. For example, olivine, a major constituent of dunite rock, is mined in several countries including Spain, Italy, Norway, Sweden, Austria, Greece, Cyprus and Turkey, and the cost price is in the order of a few tens of US$ per ton in the Rotterdam harbour. Therefore, depending on the location, an economically feasible alkaline-earth mineral can be used as the soil amendement. The world reserve of the various alkaline-earth minerals is provided by the U.S. Geological Survey in the Mineral Commodity Summaries report (Brioche, 2018).

The net cost of CO_2_ sequestration as a result of wollastonite application was estimated in our previous study (Haque et al., 2020) at $240/ton CO_2_, assuming the cost to farmer, in Ontario, of $44/ton wollastonite, and assuming a net sequestration of 0.2 t CO_2_ per ton of wollastonite, to account for CO_2_ emissions from mine to farm. The carbon price for CO_2_ emission in Ontario is expected to reach $50/ton by 2022 (Pricing carbon pollution from industry, 2019). As such, additional benefits of wollastonite, for plant growth and to replace limestone application, are important to cover the cost of wollastonite application, and more research is needed to confirm such benefits. The higher dosages of wollastonite tested in this study were intended for research purposes, to determine the upper limit of beneficial soil amendment. For real field applications, high dosages would result in higher cost of wollastonite application (in view of economic, transport and management considerations) if applied in a single season, therefore, it is more feasible to achieve such high amendment by continuously amending the soil with wollastonite every year or crop cycle.

Using a non-renewable natural resource, i.e. wollastonite in our study, in agriculture may have the limitation of reaching exhaustion in the future, though in the nearer term there are enough known reserves (and likely more) to significantly contribute to CO_2_ sequestration and agricultural benefits before significant depletion in reserves is observed. To have a continuous supply of wollastonite for mineral weathering in agriculture, wollastonite can also be synthesized chemically from limestone and silica sand (Kotsis and Balogh, 1989), a concept behind the wollastonite-containing cements of Solidia Technologies Inc. and HeidelbergCement (Canadian Wollastonite, 2019), though this is an energy intensive process and the CO_2_ released would need to be captured in geological storage sites. Additionally, there are other alkaline-earth minerals available globally that potentially can sequester CO_2_ in soils and benefit agriculture; some of these are referred to as ‘rock dust’. CO_2_ sequestration by other minerals (e.g. olivine or basalt) may not be as fast or beneficial compared to wollastonite, depending on the chemical composition and mineralogy of the rock, and further research is warranted to identify promising candidates.

### Carbon Sequestration in Soil

#### Accumulation of Inorganic Carbon in Soil

**Figure 5:**
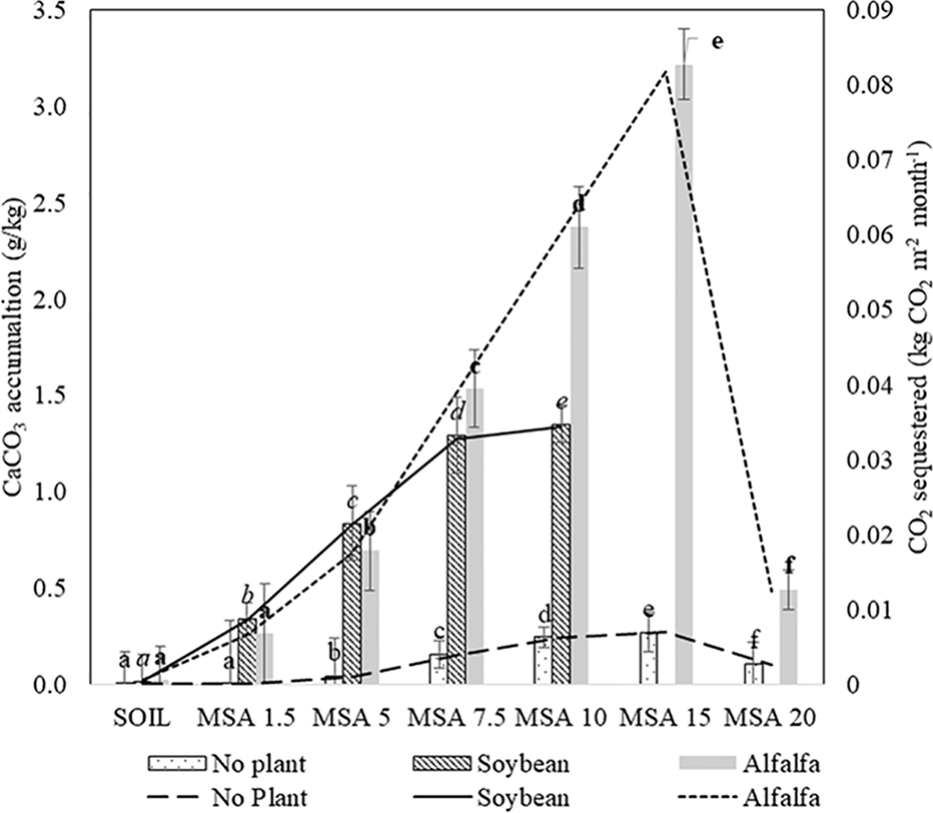
Calcium carbonate accumulation (bars) and CO_2_ sequestered (lines) in the various MSA microplots, amended with 1.5–10 wt.% wollastonite for soybean, and 1.5–20 wt.% wollastonite for alfalfa.

Overall, the inorganic carbon in the amended soil, measured in terms of calcium carbonate (CaCO_3_) content, was found to be higher than the control microplots, and the CaCO_3_ amounts that accumulated in the wollastonite-amended soils are given in **Figure 5**. In this microplot study, the plots were open to drainage to emulate the setup as close as possible to the field conditions. Based on the detailed study provided by Kelland et al. (2020), elemental mass budgets indicate that the products of basalt dissolution (alkalinity and cations) do not immediately transport directly to the marine environment *via* surface waters because of uptake of elements into plant biomass and temporary sequestration onto soil exchangeable sites (e.g. clay and organic matter). Likewise, the loss of nutrients or product of wollastonite dissolution can be assumed to have insignificant impact on the final results. For the microplots planted with soybean plants, the accumulated amount of CaCO_3_ increases with the higher dosages of wollastonite, and a similar trend is seen for the microplots planted with alfalfa up to 15 wt.% MSA application ratio, after which the inorganic carbon accumulation drastically reduced for 20 wt.% MSA. The first step of carbonate precipitation is wollastonite dissolution in soil, which is predominantly controlled by the soil pH. Geochemical modelling shows wollastonite dissolution is favored at a pH less than 10, and lower pH leads to increased wollastonite dissolution (Haque et al., 2019c). At a higher wollastonite application rate (20 wt.% MSA), there is a possibility that the soil pH resulted in slow wollastonite dissolution, thus resulting in fewer calcium ions in the soil to precipitate as carbonate. At the end of 14 weeks, the highest accumulation of 3.22 g CaCO_3_·(kg soil)^−1^ was found in the alfalfa MSA 15 microplot, which is equivalent to the sequestration of 0.3 kg CO_2_·m^−2^, for a soil depth of 0.15 m. In this study, the lowest CaCO_3_ accumulation on planted microplots occurred in the case of alfalfa grown with 1.5 wt.% MSA, which is expected and there was no significance difference with respect to the untreated soil (p > 0.05) For the remaining amended soil (MSA 5–MSA 20), the CaCO_3_ accumulation was significantly higher (p < 0.05) compare to the control soil.

With unplanted soils, the CO_2_ sequestration value did not surpass 0.025 kg CO_2_·m^−2^, reached with MSA 15, confirming the crucial role of plants, particularly leguminous, in accelerating the weathering of wollastonite in soils. Under cropped conditions, organic acids are produced from the roots, which facilitates the dissolution of the wollastonite, hence increasing the release of calcium ions in the soil, which further reacts with the dissolved CO_2_ present in the soil (as bicarbonates) to form calcium carbonate. Different types of plant species release different types of root exudates, and possess different root biomass (Nezat et al., 2004). Thus, the availability of protons and organic acids present in the soil solution for the dissolution of wollastonite will differ. This explains why the two legume species chosen in this study result in different calcium carbonate accumulation the soil, for the same amendment.

The highest calcium carbonate accumulation of 3.22 g·kg^−1^ reported in this study, obtained over 14 weeks, is equivalent to net monthly accumulation of 0.08 kg CO_2_·m^−2^·month^−1^, for a soil depth of 0.15 m. This value compares favorably to Manning et al.'s data, who reported on a plot composed of compost and quarry fines, and showed the net rate of accumulation to be in the order of 0.15 kg CO_2_·m^−2^·month^−1^ to a depth of 3 m (Manning et al., 2013). Kelland et al. (2020) report soil inorganic carbonate precipitation *via* basalt weathering in soil used to grow sorghum, reporting 0.24 kg CO_2_·m^−2^·year^−1^ (0.02 kg CO_2_·m^−2^·month^−1^) sequestration using basalt application. It is of the same order of magnitude as the rate reported in this study (0.08 kg CO_2_·m^−2^·month^−1^).

#### Mineralogical Assessment

**Figure 6:**
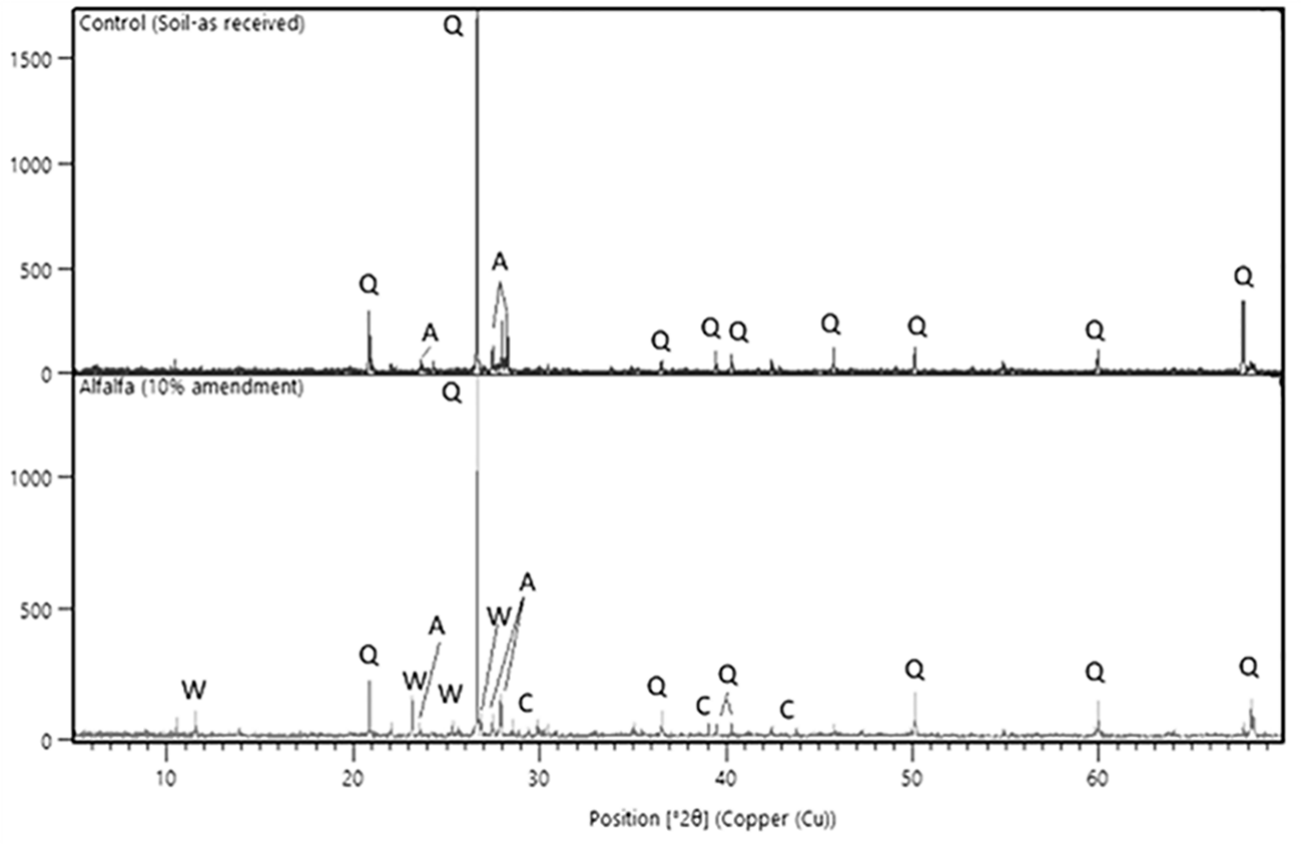
XRD diffractogram showing the main peaks and crystalline phases present in the as-received unamended soil (top) and in a wollastonite-amended soil (alfalfa MSA 10) sampled at the end of the experiment (bottom). Q, Quartz; A, Albite; W, Wollastonite; C, Calcite.

XRD analysis of the different soil samples from the wollastonite-amended microplots was conducted, and the diffractograms of two such samples are shown in **Figure 6**. Since the soil composition is complex, quantification of the different mineral phases is a challenge, therefore the peaks were qualitatively identified. The control soil showed the main peaks for quartz (SiO_2_) and albite (NaAlSi_3_O_8_), which are usually present in most of the sandy loamy soils (Schönenberger et al., 2012). The amended soil showed the characteristic peaks for wollastonite and calcite, which are not present in the control. The high calcimeter reading for amended soil is thus due to the formation of calcite, at least in part as amorphous carbonates can also form under ambient conditions (Versteegh et al., 2017).

#### SEM-EDS Study

**Figure 7:**
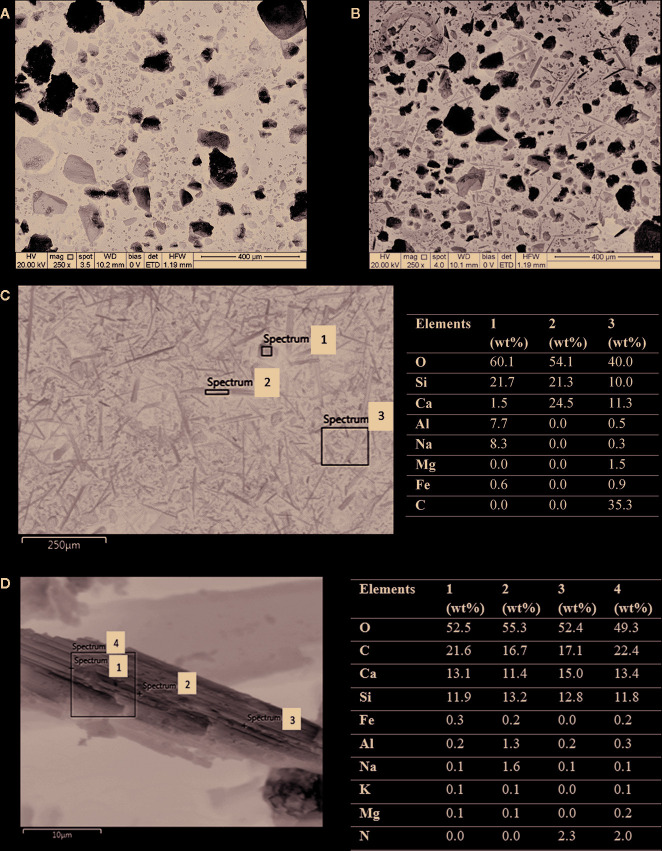
SEM images of **(A)** soil (control), and **(B)** wollastonite-amended soil at the end of the experiment (alfalfa MSA 10). SEM-EDS of **(C)** fine wollastonite (as-received) and of **(D)** carbonated wollastonite.

**Figures 7A, B** show SEM images of the control (soil) and a wollastonite-amended soil sampled at the end of the experiment, respectively. The fine needle-shaped wollastonite is noticeable in the amended soil sample (**Figure 7B**). A closer look at the EDS spectrum of the carbonated wollastonite fragments reveals the chemical profile. Comparing the EDS data of the uncarbonated wollastonite with the carbonated sample provides information on the occurrence of weathering and mineral carbonation. **Figure 7C** shows the SEM-EDS data for the as-received wollastonite used in this study. The needle-shaped structure (**Figure 7C**, spectrum 2) contains silicon (Si), calcium (Ca), and oxygen (O) as the main elements. An irregular-shaped structure (**Figure 7C**, spectrum 1) consists of Si, Ca, and O, which are the main elements present in wollastonite, as well as Mg, Na, Fe, and Al, which are usually present in trace amounts. **Figure 7D** shows the SEM-EDS data for a carbonated wollastonite fragment from the amended soil. Spot EDS analysis at four different points shows that this fragment consists of Ca, C, Si, and O as the main elements, suggesting that the formation of CaCO_3_. The Si content of this fragment at various points is in the range of 11.8–13.2 wt.%, which is lower than that of the feedstock wollastonite (**Figure 7C**) containing approximately 21 wt.% Si. This implies that wollastonite dissolution (Equation (2)) has taken place, resulting in the release of Si during the enhanced weathering reactions. The presence of the C content on the wollastonite grain confirms that carbonation occurred, and carbonate is precipitated on the wollastonite surface.

**Figure S8** shows SEM image of other mineral grains from amended soil, this time associated with organic matter. The elemental composition of these two fragments (Spectrums 1 and 3) identifies Ca, Si, and O as the main elements, which are characteristic for quartz (**Figure S8A**) wollastonite (**Figure S8B**), along with traces of Mg, P, K, Al, Fe, as well as C, here representing the organic carbon. Organic carbon identification is made by looking at the morphology of the material attached to the mineral fragments, as well as the presence of trace elements typical for organic matter, which were not seen in the carbonated wollastonite (**Figure 7D**).

## Conclusion

The results of this study are of significance for climate change mitigation *via* wollastonite weathering in soil. This study has demonstrated that following 14 weeks of exposure to ambient Ontario atmospheric conditions, inorganic carbonate accumulated in the wollastonite-amended soil, and CO_2_ sequestration of 0.3 kg CO_2_·m^−2^, for a soil depth of 0.15 m, was achieved. Calcite was observed in the XRD diffractogram, and dissolution of wollastonite was evident from the SEM-EDS study. Wollastonite amendment of soils also resulted in better growth of soybean and alfalfa plants, as indicated by the biomass dry weight yield data, thus implying the positive effect of wollastonite on agricultural crops and its potential to be used as a soil amendment in the agricultural sector. These co-benefits of wollastonite soil amendment would encourage producers to effectively use wollastonite to contribute towards global climate change mitigation, without compromising their produce. Findings from this microplot study serve to guide future field scale studies, now that we know the optimal and, even more importantly, the limiting dosages that lead to good plant growth. To further elucidate the effect of wollastonite on plant growth, study on the transport of silicon in the plants and the accumulation of phytogenic silica in the different plant tissues (shoots, roots, as well as leaves) will be insightful. A greenhouse-based experiment on these topics has been initiated in our research group, which also aims to study the mutual action when wollastonite is combined with conventional fertilizers and liming agents. Opportunity also exists to investigate the benefits of wollastonite amendment for crops under stressed conditions, including in urban rooftop/balcony farms, and to isolate the physical effects of wollastonite amendment in soils from the chemical and (micro)biological effects. Such thorough studies will provide a deeper understanding on the effect of wollastonite application as a soil amendment.

## Data Availability Statement

The raw data supporting the conclusions of this article will be made available by the authors, without undue reservation.

## Author Contributions

FH: Methodology, Formal analysis, Investigation, Writing—Original Draft. RS: Conceptualization, Methodology, Writing—Review and Editing, Supervision, Funding acquisition. YC: Conceptualization, Methodology, Resources, Supervision, Project administration, Funding acquisition.

## Funding

This research was financially supported by Low Carbon Innovation Fund from the Ministry of Research Innovation and Science (Ontario, Canada).

## Conflict of Interest

The authors declare that the research was conducted in the absence of any commercial or financial relationships that could be construed as a potential conflict of interest.
