# Supplementary material for: Optimizing Inorganic Carbon Sequestration and Crop Yield With Wollastonite Soil Amendment in a Microplot Study
Source: Front Plant Sci. 2020 Jul 3;11:1012. doi: 10.3389/fpls.2020.01012 (PMC7350405; doi:10.3389/fpls.2020.01012)
Supplement: Supplementary file 1 [file DataSheet_1.pdf]

Supplementary Material

Microplot set up data

The rooftop experiment was conducted in Guelph, ON, Canada. However, the nearest historical weather data available was located on Kitchener/Waterloo (20 km distance), as shown in Figures S1a and S1b.

a)

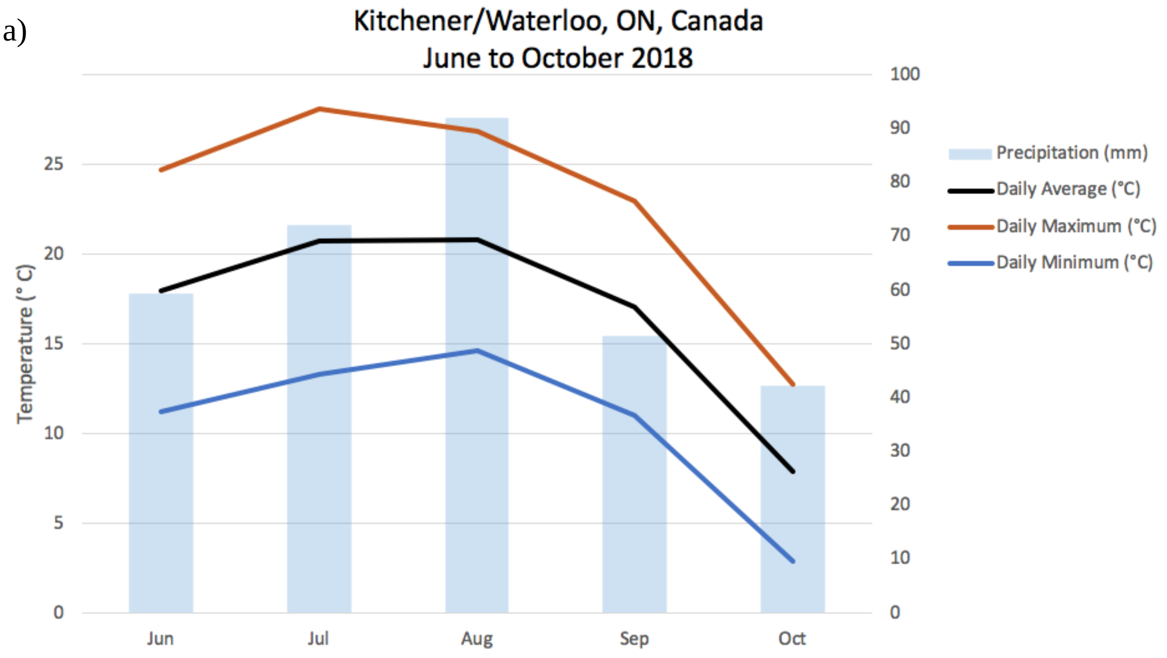

b)

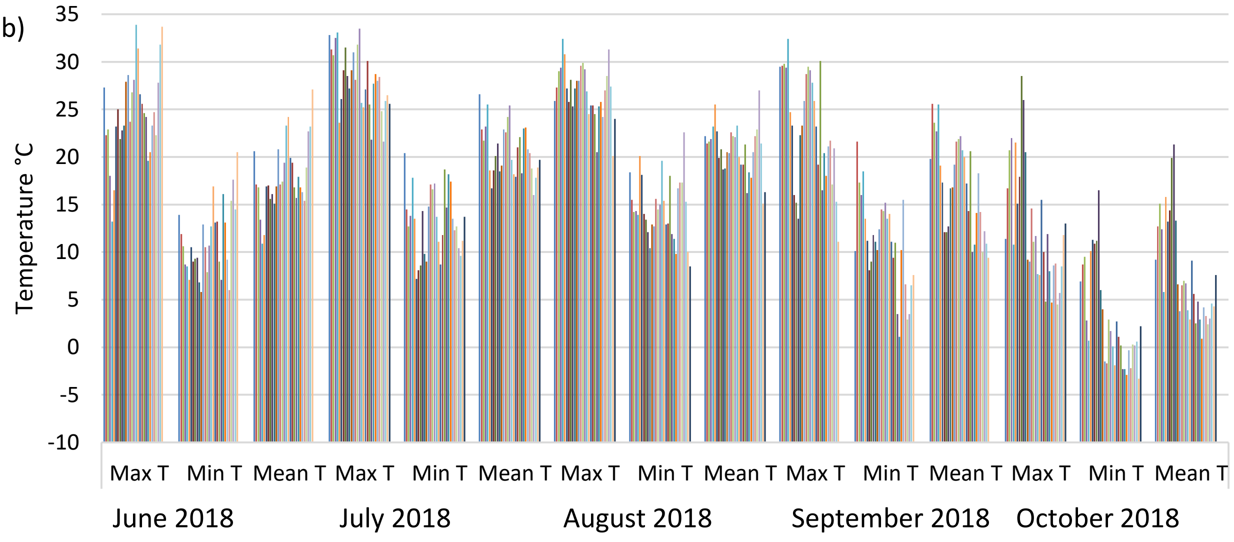

c)

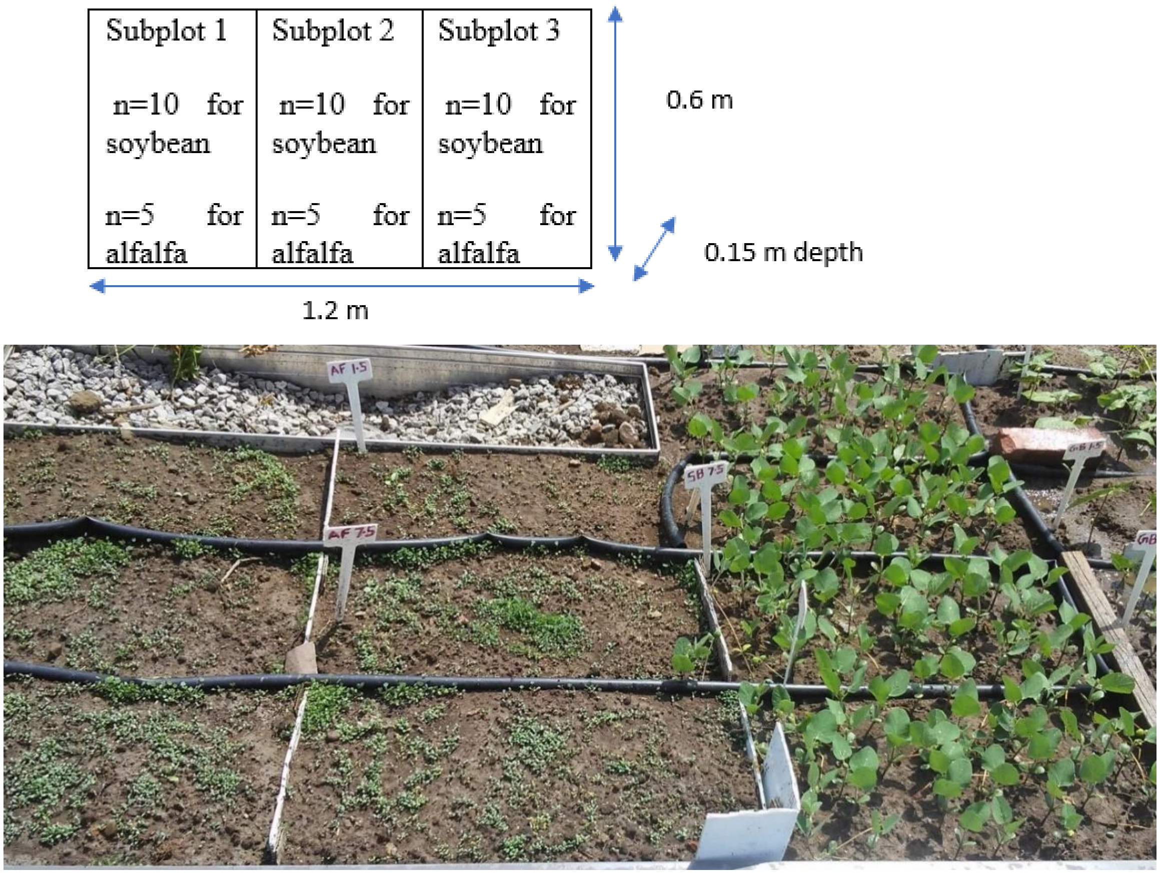

Figure S1. a) Kitchener/Waterloo, ON (Canada) weather historical data from June to October of 2018 during the experiment (Environment and Climate Change Canada, 2019) (Reused with permission: CC BY 4.0 (Dudhaiya et al., 2019)). b) Daily maximum, minimum, and mean temperature data June to October of 2018 during the experiment (Environment and Climate Change Canada, 2019). c) Microplot set up and a sample picture of the plots.

Figure S1c shows the experimental design. Each microplot has a dimension of 0.6 m length, 1.2 m width, and 0.15 m depth, and each subplot represents one-third of the microplot. For the soybean, 10 soybean seeds were sowed, in rows, per subplot, and for alfalfa the seeding rate was 1.3 g/m^2. Since the alfalfa seeds are very small, the spreading was done on mass basis, as practiced in the fields, in a random order. For the plant analysis, all the soybean plants were harvested and analyzed (n=30 for the microplot or n=10 for each subplot). In case of alfalfa, there were numerous plants, therefore 5 plants per subplot was chosen for analysis (n=15 for each microplot). For soybean, yield is also determined, therefore all the plants were harvested.

Additional materials and samples data

Elemental composition of the soil was quantified by Wavelength Dispersive X-Ray Fluorescence (WDXRF, Malvern Panalytical Zetium). Duplicate samples, in loose powder form, were analyzed for 20 minutes using standardless Omniam method, under helium and at 1 kW power, and concentrations were calculated as oxides. The average sum before normalization was 70.7 wt%, with the balance being porosity and undetectable light elements (H, C, O, N). The average concentrations of the detected oxides present in amounts greater than 0.10 wt%, normalized to 100%, in decreasing order, were: 65.06 wt% SiO_2; 14.71 wt% Al_2O_3; 6.27 wt% Fe_2O_3; 3.62 wt% CaO; 3.03 wt% K_2O; 2.02 wt% P_2O_5; 1.81 wt% MgO; 1.51 wt% Na_2O; 1.19 wt% TiO_2; 0.34 wt% SO_3; 0.15 wt% MnO; 0.10 BaO; 0.10 wt% ZrO_2.

Table S1: Soil characteristics

pH	6.63
Dry organic matter	32 g/kg
Soil texture	Sandy Loam
Gravel	11 g/kg
Sand	551 g/kg
Silt	295 g/kg
Clay	155 g/kg
XRF	
SiO_2	65.06 wt%
Al_2O_3	14.71 wt%
Fe_2O_3	6.27 wt%
CaO	3.62 wt%
K_2O	3.03 wt%
P_2O_5	2.02 wt%
MgO	1.81 wt%
Na_2O	1.51 wt%
TiO_2	1.19 wt%
SO_3	0.34 wt%
MnO	0.15 wt%
BaO	0.10 wt%
ZrO_2	0.10 wt%
Malvern Panalytical Zetium Specifications:	Elemental range of Na - Am.
	Concentration range of ppm - 100 wt%.
	High count rate capability of up to 1 Mcps
	Spot size of 0.5 mm.
	Stepwise positioning of 100 µm

Table S2: Soybean stem width and leaf blade width with different dosages of wollastonite amendment. In all cases, n = 30 and the uncertainties are the standard deviation.

	**Soil (Control)**	**MSA 1.5**	**MSA 5**	**MSA 7.5**	**MSA 10**
Stem width (mm)	3.2 ± 0.3	3.0 ± 0.3	3.1 ± 0.3	3.0 ± 0.4	3.1 ± 0.4
Leaf blade (mm)	29.8 ± 3.6	31.0 ± 4.2	32.3 ± 3.3	30.1 ± 4.2	30.1± 4.2

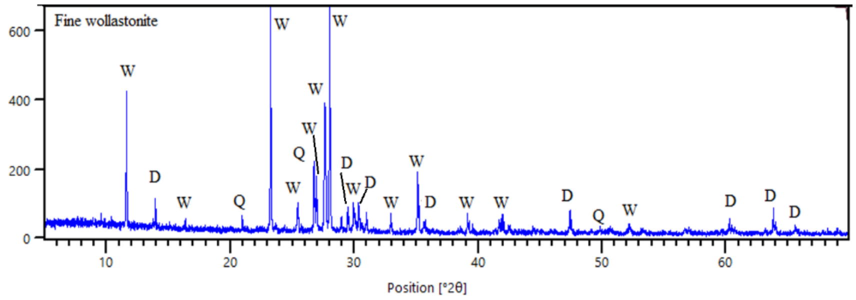

Figure S2. XRD diffractogram of the wollastonite, determined for to identify the major mineral phases. (W: Wollastonite, D: Diopside, Q: Quartz).

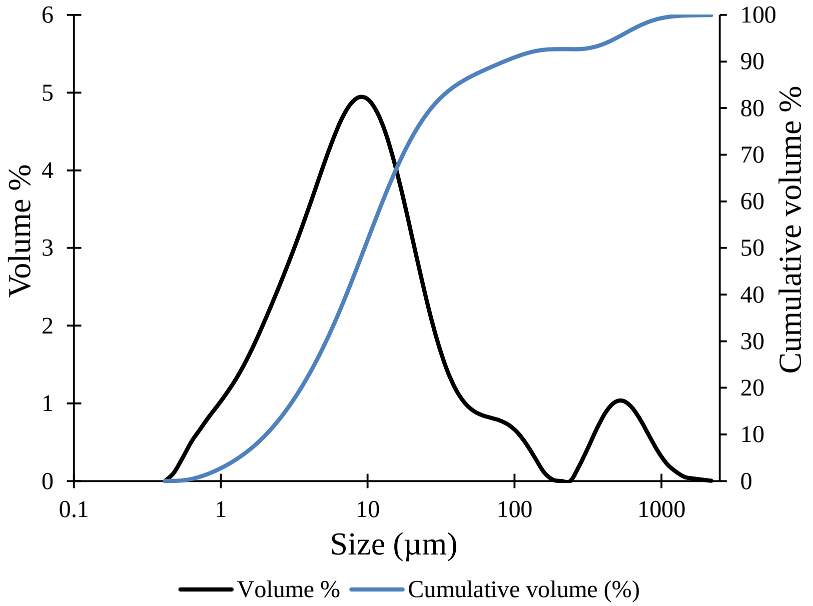

Figure S3. Particle size distribution of Canadian wollastonite, determined by wet laser diffraction.

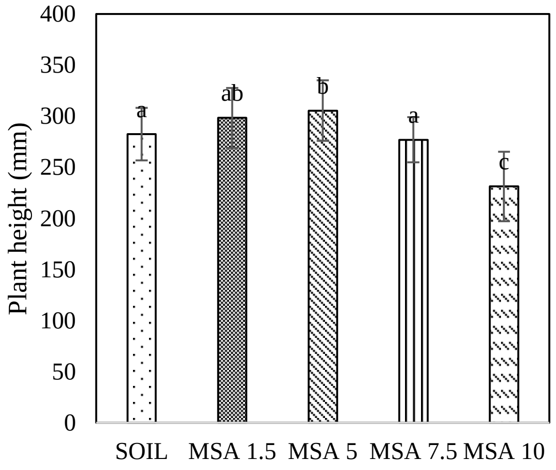

Figure S4. Variation in the soybean plant height with different dosages (wt% in soil) of wollastonite mineral soil amendment (MSA). In all cases, n = 30 and statistically analyzed using Tukey test.

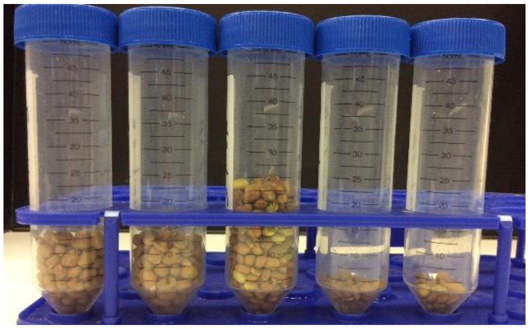

Figure S5. Soybean seeds produced from each microplot (30 plants) with different dosages of wollastonite amendment (left to right: SOIL, MSA 1.5, MSA 5, MSA 7.5, MSA 10).

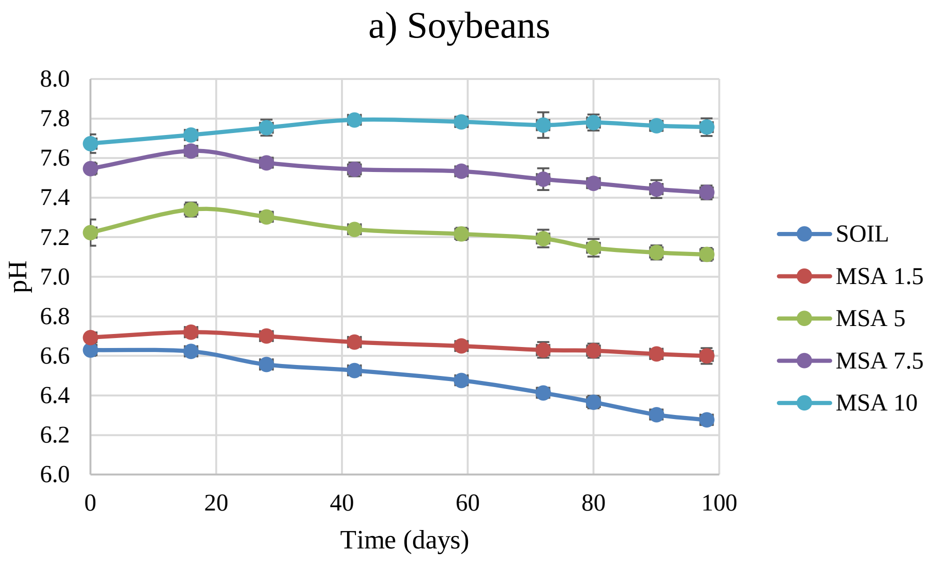

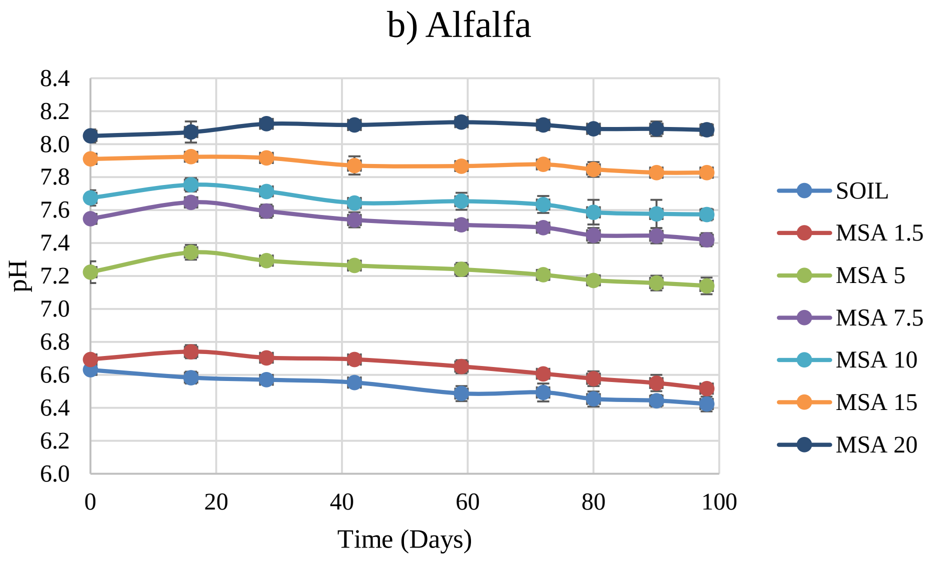

Figure S6. Variation of pH with time of various wollastonite-amended soils grown with a) soybeans and b) alfalfa.

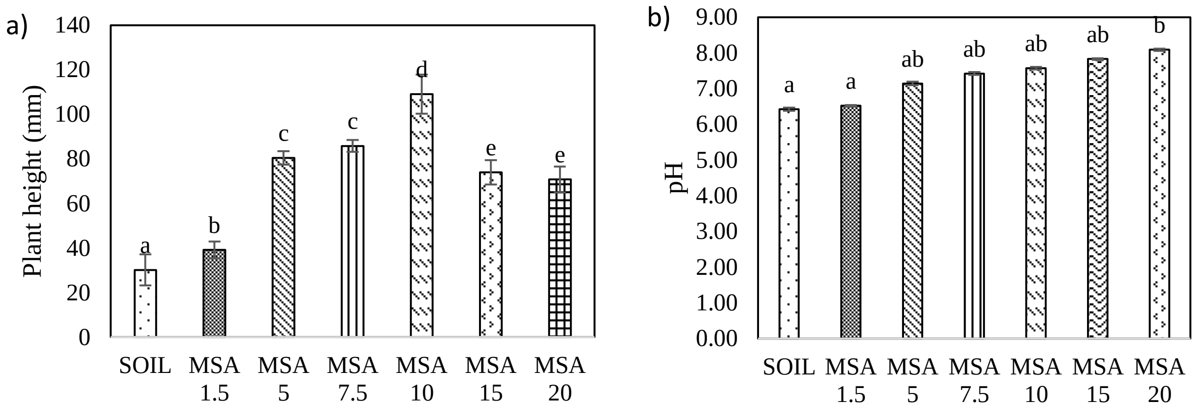

Figure S7. Variation in the alfalfa: a) plant height and b) pH of the soil at the end of the experiments, with different dosages (wt% in soil) of wollastonite mineral soil amendment (MSA).

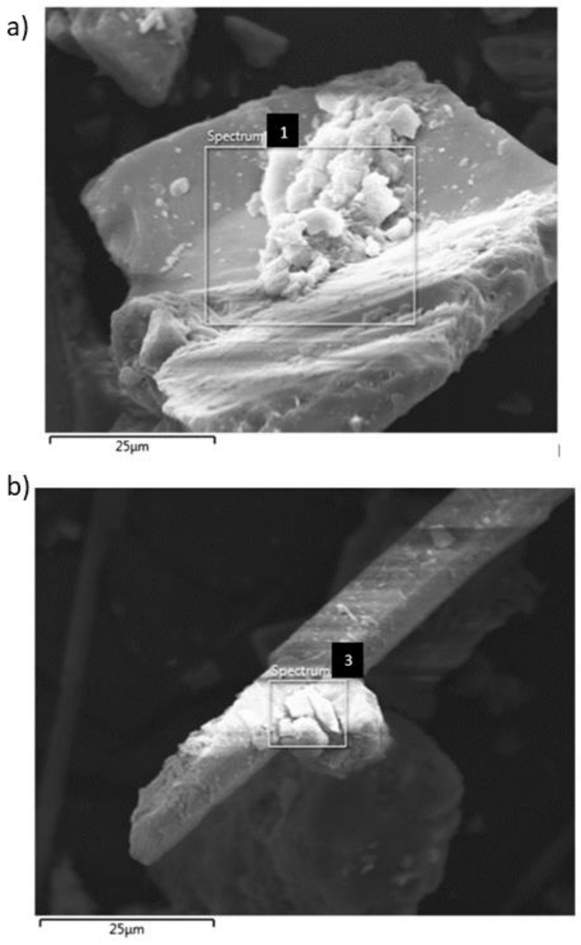

Elements	1 (wt%)	3 (wt%)
O	53.8	53.9
C	22.0	16.6
Ca	0.5	8.7
Si	18.4	10.8
Fe	1.6	0.9
Al	2.6	0.7
Na	0.2	0.1
K	0.4	0.2
Mg	0.5	1.0
N	0.0	0.0

Figure S8. SEM images of mineral fragments from soil amended with wollastonite: a) quartz associated with organic matter; and b) needle shaped wollastonite associated with organic matter.

Measured Data:

Soybean root dry biomass (g)

n	SOIL	MSA 1.5	MSA 5	MSA 7.5	MSA 10
1	0.3341	0.338	0.5534	0.3588	0.33
2	0.2864	0.3843	0.5313	0.3997	0.2025
3	0.2974	0.3912	0.3036	0.1898	0.1054
4	0.2546	0.3507	0.3196	0.3962	0.27
5	0.3214	0.3893	0.4888	0.3453	0.3679
6	0.3364	0.2877	0.5029	0.5205	0.4843
7	0.3647	0.4153	0.324	0.3221	0.1844
8	0.4001	0.35	0.4581	0.381	0.2559
9	0.4214	0.3192	0.588	0.379	0.332
10	0.1845	0.3797	0.2262	0.2668	0.3616
11	0.1687	0.3645	0.495	0.2909	0.2447
12	0.2485	0.259	0.2323	0.4094	0.3602
13	0.3147	0.3322	0.4407	0.2015	0.1883
14	0.2436	0.3254	0.3498	0.3715	0.2475
15	0.2143	0.3184	0.399	0.3259	0.2864
16	0.4375	0.3743	0.6247	0.3307	0.1984
17	0.0945	0.7554	0.4804	0.2894	0.2347
18	0.1157	0.3382	0.4022	0.2739	0.3475
19	0.3124	0.3972	0.3178	0.469	0.3314
20	0.2894	0.4892	0.3484	0.3302	0.3006
21	0.2657	0.3845	0.4524	0.2478	0.2089
22	0.3125	0.3957	0.3393	0.3582	0.1974
23	0.4397	0.4287	0.3391	0.275	0.2047
24	0.3657	0.4675	0.4218	0.4442	0.2675
25	0.3842	0.5002	0.4417	0.354	0.2849
26	0.3579	0.4895	0.3221	0.1763	0.2478
27	0.3389	0.3489	0.4046	0.2491	0.3067
28	0.3984	0.3984	0.4856	0.2975	0.2947
29	0.3674	0.4007	0.4011	0.3211	0.1984
30	0.4321	0.4127	0.3357	0.3014	0.0945

Soybean above-ground dry biomass (g)

n	SOIL	MSA 1.5	MSA 5	MSA 7.5	MSA 10
1	2.3053	4.6062	8.9807	2.5371	2.0883
2	3.5233	4.8753	10.7135	3.6661	1.7608
3	2.8724	5.1407	7.5693	1.9973	1.7766
4	2.5567	2.4766	6.1647	3.2214	2.2014
5	3.6845	3.3	5.1416	2.9095	5.4114
6	3.1604	5.8708	12.7343	3.6932	4.1287
7	3.912	5.2051	5.7003	2.5873	1.6374
8	2.1674	2.5248	6.2094	2.9357	2.3415
9	3.9435	2.933	5.6774	2.4958	3.3317
10	3.6451	3.8175	5.2258	2.2567	4.0875
11	3.1347	2.7805	5.5107	2.6513	5.6314
12	4.0614	3.985	7.1945	3.9084	3.4783
13	4.8531	2.7364	6.7875	1.3216	4.5712
14	2.9066	2.8226	7.9924	3.2244	1.2485
15	3.9429	2.1368	6.6392	2.8584	1.3478
16	3.8472	3.2064	5.6918	2.2677	1.6342
17	3.4024	4.4626	8.9086	3.2414	1.6287
18	3.6782	4.6084	5.0134	2.4804	2.2475
19	3.4736	4.8103	5.7048	1.3569	2.3945
20	3.9974	4.4832	8.6298	4.1038	4.1257
21	3.6843	4.6134	6.7058	9.3009	3.0745
22	2.8746	5.8762	5.0554	2.6014	3.3486
23	4.0214	5.5537	6.4702	3.5768	2.4128
24	2.9976	3.8947	8.7242	4.0254	3.1284
25	2.8463	2.9872	6.5666	6.1999	3.6241
26	3.5796	4.4437	5.9019	4.3178	3.302
27	3.9987	3.6843	6.303	5.8745	4.0578
28	4.0265	3.9965	7.2141	4.0367	4.1265
29	4.5361	3.8763	6.2598	3.9876	3.9475
30	4.8927	4.0221	11.5476	5.8756	4.8751

Soybean plant height (mm)

n	SOIL	MSA 1.5	MSA 5	MSA 7.5	MSA 10
1	268.09	332.19	295.36	298.42	248.58
2	255.46	316.92	255.6	302.17	250.96
3	293.05	282.81	280.65	258.12	253.35
4	284.64	307.53	302.71	278.94	206.78
5	267.93	267.76	267.38	275.74	257.49
6	271.4	303.27	247.89	256.27	199.06
7	282.36	303.27	280.27	306.15	263.22
8	231.48	282.59	299.01	242.43	238.02
9	237.59	305.06	302.45	298.85	240.1
10	252.73	300.34	298.63	278.39	258.25
11	309.24	282.17	306.26	250.28	226.99
12	314.86	287.71	284.42	288.93	255.77
13	289.96	309.21	301.77	226.51	221.02
14	278.24	276.79	286.05	309.24	231.38
15	290.92	269.95	306.92	263.46	258.5
16	274.94	381.27	280.31	275.17	193.71
17	287.9	304.17	329.12	248.31	172.6
18	297.23	262.75	328.11	268.07	270.76
19	309.24	300.64	321.87	302.61	266.33
20	299.11	358.75	337.15	283.56	175.71
21	291.22	276.06	362.1	282.19	265.23
22	329.24	289.32	327.38	266.93	261.85
23	284.99	330.5	309.24	286.03	172.21
24	295.2	342.3	309.24	300.04	244.22
25	284.75	286.99	312.85	260.99	278.41
26	206.47	290.73	314.25	264.57	257.34
27	296.12	242.73	298.67	323	219.75
28	295.14	267.19	279.86	267.42	185.99
29	299.18	286.18	363.45	266.98	176.84
30	297.36	301.54	374.86	279.56	186.84

Soybean stem width (mm)

n	SOIL	MSA 1.5	MSA 5	MSA 7.5	MSA 10
1	3.46	3.3	3.34	2.61	2.82
2	3.9	3.27	3.41	3.4	2.35
3	3.21	2.6	2.89	3.86	3.88
4	2.42	3.54	3.37	2.79	3.05
5	3.16	2.79	2.95	2.88	3.43
6	3.24	2.69	2.78	2.77	3.49
7	3.22	3.32	2.99	3.36	2.89
8	3.21	2.59	3.35	3.57	3.51
9	3.1	2.86	3.53	2.2	2.76
10	2.85	2.7	3.17	2.66	3.18
11	3.12	2.82	3.13	2.9	3.28
12	3.45	2.44	2.84	2.84	3.24
13	2.73	3.17	3.12	2.91	3.33
14	3.12	2.67	2.92	3.45	3.34
15	3.25	3.01	2.75	3.42	2.72
16	3.36	3.15	3.74	3.34	3.29
17	2.97	3.25	2.53	2.65	3.51
18	3.16	3.14	2.94	2.34	3.52
19	2.69	3.2	3.29	2.76	3.48
20	2.74	2.9	2.76	2.96	3.14
21	3.44	2.67	2.52	2.99	1.84
22	3.21	3.36	2.96	3.11	3.5
23	3.23	3.41	2.98	3.35	2.9
24	2.85	3.36	3.06	2.69	3.49
25	3.49	3.4	2.93	2.34	3.21
26	3.7	3.42	3.14	3.12	2.89
27	3.45	2.98	3.43	3.81	2.45
28	3.28	2.86	3.35	2.87	3.31
29	2.94	2.75	3.07	3.27	3.05
30	2.78	2.68	2.74	3.12	2.99

Soybean leaf blade width (mm)

n	SOIL	MSA 1.5	MSA 5	MSA 7.5	MSA 10
1	34.26	19.48	34.47	24.11	30.93
2	32.11	25.49	37.11	32.31	28.41
3	29.27	32.52	42.51	32.77	24.09
4	35.53	37.43	29.57	28.98	33.82
5	33.07	36	38.13	34.01	31.29
6	30.63	30.73	26.02	32.51	34.82
7	29.25	32.05	28.16	33.13	28.63
8	30.94	29.41	33.04	27.33	24.95
9	27.33	27.62	33.71	18.75	31.34
10	26.27	30.17	31.96	30.15	35.99
11	25.23	32.72	31.58	29.53	28.5
12	31.68	26.94	29.71	30.94	32.59
13	28.37	37.39	35.33	30.26	38.26
14	33.16	24.77	30.27	22.76	25.07
15	29.93	32.48	30.13	33.39	29.04
16	28.92	32.42	32.32	29.96	22.06
17	37.62	30.56	31.63	31.96	31.06
18	27.35	31.73	30.44	24.36	27.59
19	33.37	31.78	30.54	36.99	32.89
20	30.62	32.39	31.63	24.26	21.21
21	23.82	33.11	33.51	31.78	39.15
22	31.4	27.19	36	30.09	29.77
23	22.56	38.26	29.06	30.66	31.77
24	23.3	31.35	33.83	24.14	26.74
25	26.75	31.54	31.65	33.63	33.12
26	31.05	32.75	29.23	33.05	29.84
27	29	29.84	35.17	31.05	30.64
28	26.41	27.55	33.34	33.48	27.36
29	33.27	37.64	29.58	30.87	31.45
30	30.89	26.48	30.48	36.87	31.84

Soybean yield (g)

Set	SOIL	MSA 1.5	MSA 5	MSA 7.5	MSA 10
Subplot 1	5.4184	4.9475	10.1726	2.0145	1.0945
Subplot 2	6.1243	5.0126	11.3704	1.9245	2.0145
Subplot 3	3.875	5.8971	10.001	1.5551	0.8749

Alfalfa root dry biomass (g)

n	SOIL	MSA 1.5	MSA 5	MSA 7.5	MSA 10	MSA 15	MSA 20
1	0.0287	0.0274	0.0336	0.0502	0.0531	0.0556	0.0329
2	0.0328	0.0306	0.0399	0.0489	0.0549	0.0533	0.0422
3	0.0219	0.0254	0.0224	0.053	0.0715	0.0446	0.0388
4	0.0221	0.0173	0.0274	0.0588	0.0828	0.0488	0.0417
5	0.0378	0.0339	0.00282	0.057	0.0533	0.0488	0.0546
6	0.0261	0.0196	0.0273	0.0402	0.075	0.0561	0.0503
7	0.0286	0.0329	0.0389	0.0413	0.089	0.0457	0.0447
8	0.0136	0.0197	0.0244	0.0481	0.088	0.0732	0.0473
9	0.0229	0.0344	0.0256	0.0475	0.0684	0.0533	0.0504
10	0.0212	0.0113	0.0267	0.0439	0.0655	0.073	0.0402
11	0.023	0.0245	0.0344	0.0547	0.0641	0.0624	0.0429
12	0.0193	0.0264	0.0314	0.0489	0.0509	0.0846	0.0389
13	0.0112	0.0302	0.0309	0.0521	0.0542	0.0486	0.0202
14	0.0124	0.0292	0.0282	0.0471	0.0692	0.0509	0.0281
15	0.0189	0.0242	0.03	0.0503	0.0614	0.0453	0.0408

Alfalfa above-ground dry biomass (g)

n	SOIL	MSA 1.5	MSA 5	MSA 7.5	MSA 10	MSA 15	MSA 20
1	0.0268	0.0503	0.0923	0.1238	0.1045	0.1079	0.0745
2	0.0449	0.0673	0.0806	0.1043	0.0994	0.0986	0.1329
3	0.0421	0.0305	0.0533	0.1011	0.1084	0.0899	0.0751
4	0.0287	0.0338	0.0612	0.0913	0.1348	0.0788	0.0931
5	0.0446	0.0515	0.0683	0.0811	0.1133	0.0933	0.0855
6	0.0220	0.0342	0.0625	0.0863	0.1318	0.1461	0.1321
7	0.0271	0.0481	0.0554	0.097	0.1715	0.0965	0.0738
8	0.0200	0.0248	0.0669	0.1054	0.1612	0.1219	0.0704
9	0.0567	0.0529	0.0684	0.1139	0.1251	0.1749	0.0993
10	0.0306	0.0334	0.0953	0.1539	0.1074	0.1414	0.0854
11	0.0444	0.0469	0.0662	0.1109	0.145	0.1044	0.0879
12	0.0576	0.0301	0.0828	0.0989	0.1001	0.0874	0.0712
13	0.0218	0.0622	0.0609	0.0942	0.159	0.1067	0.0863
14	0.0243	0.0618	0.0659	0.0943	0.1277	0.1086	0.0873
15	0.0293	0.0391	0.061	0.0987	0.0907	0.1088	0.0784

Alfalfa plant height (mm)

n	SOIL	MSA 1.5	MSA 5	MSA 7.5	MSA 10	MSA 15	MSA 20
1	31.01	31.61	84.03	84.76	96.71	76.20	62.01
2	46.96	40.60	84.39	86.47	116.99	77.89	73.25
3	18.11	39.03	82.47	87.24	120.45	83.46	73.43
4	27.69	35.62	81.15	89.10	102.88	84.79	75.29
5	27.56	41.25	77.13	81.24	105.97	71.99	66.07
6	33.56	36.79	80.53	84.57	114.69	74.25	79.94
7	27.89	41.31	77.02	88.95	100.15	73.45	76.46
8	22.59	44.97	80.23	82.45	105.90	79.24	67.54
9	36.57	39.27	78.39	81.72	102.98	70.64	76.46
10	28.58	44.10	83.14	84.96	98.82	70.46	61.54
11	25.83	43.09	76.16	85.39	115.45	69.89	62.41
12	33.00	33.24	78.02	85.59	112.34	73.14	70.24
13	37.63	36.88	79.96	88.56	100.98	68.79	71.25
14	31.75	40.05	86.14	85.62	117.84	65.12	69.85
15	23.79	39.84	78.26	89.74	124.16	70.34	75.42

Calcimetry results

Amendment	No plant	Soybean	Alfalfa
SOIL	0.009	0.018	0.026
MSA 1.5	0.003	0.344	0.264
MSA 5	0.044	0.838	0.694
MSA 7.5	0.159	1.291	1.535
MSA 10	0.246	1.351	2.372
MSA 15	0.274	-	3.216
MSA 20	0.106	-	0.493

References:

Environment and Climate Change Canada. (2019). http://climate.weather.gc.ca/climate_data/hourly_data_e.html?hlyRange=2010-04-06%7C2019-09-07&dlyRange=2010-04-18%7C2019-09-07&mlyRange=%7C&StationID=48569&Prov=ON&urlExtension=_e.html&searchType=stnName&optLimit=yearRange&StartYear=1840&EndYear=2019&selRowPerPage=25&Line=0&searchMethod=contains&txtStationName=waterloo&timeframe=1&Year=2018&Month=6&Day=1# [Accessed September 7, 2019).

Dudhaiya, A., Haque, F., Fantucci, H., & Santos, R. M. (2019). Characterization of Physically Fractionated Wollastonite-Amended Agricultural Soils. Minerals. 9(10): 635. doi: 10.3390/min9100635
